# Dose adjustment not required for contezolid in patients with moderate hepatic impairment based on pharmacokinetic/pharmacodynamic analysis

**DOI:** 10.3389/fphar.2023.1135007

**Published:** 2023-03-13

**Authors:** Junzhen Wu, Xinyi Yang, Jufang Wu, Jingjing Wang, Hailan Wu, Yu Wang, Hong Yuan, Huahui Yang, Hailin Wang, Jing Zhang

**Affiliations:** ^1^ Phase I Unit, Huashan Hospital, Fudan University, Shanghai, China; ^2^ Institute of Antibiotics, Huashan Hospital, Fudan University, Shanghai, China; ^3^ Key Laboratory of Clinical Pharmacology of Antibiotics, National Health and Family Planning Commission, Shanghai, China; ^4^ National Clinical Research Center for Aging and Medicine, Huashan Hospital, Fudan University, Shanghai, China; ^5^ Shanghai MicuRx Pharmaceutical Co., Ltd., Shanghai, China

**Keywords:** contezolid, hepatic impairment, dose adjustment, safety, dosing regimen, pharmacokinetic/pharmacodynamic analysis

## Abstract

**Objective:** Contezolid is an oxazolidinone antimicrobial agent newly approved for treatment of Gram-positive bacterial infections. It is primarily metabolized by the liver. This study aimed to assess whether it is required to adjust the dose of contezolid in patients with moderate hepatic impairment for clinicians to use the drug more rationally.

**Methods:** A single-center, open-label, parallel-group study was conducted to compare the pharmacokinetic (PK) parameters of contezolid and its metabolite M2 between the patients with moderate hepatic impairment and healthy controls with normal liver function after oral administration of 800 mg contezolid tablets. Monte Carlo simulation was performed to calculate the probability of target attainment (PTA) and cumulative fraction of response (CFR) of contezolid based on the PK and pharmacodynamic data.

**Results:** Oral treatment with 800 mg contezolid tablets was safe and well tolerated in both the patients with moderate hepatic impairment and healthy controls. Moderate hepatic impairment did not result in substantial difference in the area under the concentration-time curve from 0 to 24 h (AUC_0–24h_, 106.79 vs. 97.07 h μg/mL) of contezolid even though lower maximum concentration (C_max_, 19.03 vs. 34.49 μg/mL) compared with healthy controls. The mean cumulative amount excreted in urine from 0 to 48 h (Ae_0–48h_) and renal clearance (CL_R_) of contezolid did not show significant difference between the two groups. Moderate hepatic impairment was associated with lower C_max_, slightly lower AUC and Ae_0–48h_ of M2 compared to the healthy controls. *f*AUC/MIC was the best PK/PD index to predict the clinical efficacy of contezolid. Monte Carlo simulation results indicated that at the proposed *f*AUC/MIC target value of 2.3, the dosing regimen of oral contezolid 800 mg q12h could achieve satisfactory PTA and CFR (both >90%) for the target pathogen (methicillin-resistant *S. aureus*, MIC ≤4 mg/L) in patients with moderate hepatic impairment.

**Conclusion:** Our preliminary data suggest that dose adjustment is not required for contezolid in patients with moderate hepatic impairment.

**Clinical Trial Registration:** https://chinadrugtrials.org.cn, identifier: CTR20171377.

## 1 Introduction

The infections caused by methicillin-resistant *Staphylococcus aureus* (MRSA), such as bloodstream infection, endocarditis, bone and joint infection, and skin and soft tissue infection, are usually associated with high morbidity and mortality (Turner et al., 2019; Johnson et al., 2021). According to China Antimicrobial Surveillance Network (CHINET), the average prevalence of MRSA was 31% in 2020 in the hospitals across China (Fupin et al., 2021).

Vancomycin has been the first choice and sometimes the last resort for treatment of serious MRSA infections (Lakhundi and Zhang, 2018). However, nephrotoxicity and emerging resistance have limited its clinical use. Linezolid, as the first oxazolidinone antibiotic available in clinical practice, is effective for managing the infections caused by MRSA (Rodvold and McConeghy, 2014) or vancomycin-resistant *S. aureus* (VRSA). However, longer treatment duration (>2 weeks) is linked to increased incidence of myelosuppression and other side effects (Gerson et al., 2002).

Contezolid (formerly known as MRX-I) is another oxazolidinone antibiotic newly approved in 2021 for treatment of complicated skin and soft tissue infections (cSSTIs) in China (Hoy, 2021). It has potent activity against Gram-positive bacteria, particularly the resistant strains such as MRSA, methicillin-resistant *Staphylococcus epidermidis* (MRSE), penicillin-resistant *Streptococcus pneumoniae* (PRSP), and vancomycin-resistant *Enterococcus* (VRE) (Wu et al., 2020). Contezolid has a more favorable safety profile than linezolid in terms of myelosuppression and monoamine oxidase inhibition (Wu et al., 2020). The results of phase I studies in China and Australia have indicated that contezolid was rapidly absorbed after oral single dose administration. Plasma concentration reached peak about 2 h post dose. The maximum plasma concentration (C_max_) and the area under the plasma concentration-time curve (AUC) increased with dose, but the increase was not linear for doses above 800 mg (Eckburg et al., 2017; Wu et al., 2018). Contezolid was metabolized mostly by the liver, involving the oxidative opening of the dihydropyridine (DHPO) ring (Meng et al., 2015). About 0.8%–2.3% of the administered dose of contezolid was excreted in unchanged form *via* kidneys within 48 h after single-dose or multiple-dose administration (Wu et al., 2018).

A mass balance study in humans with a single oral dose [^14^C]contezolid reported that 91.5% of the administered dose was recovered from urine (76.7% of the dose) and feces (14.8% of the dose) over a 168-h period after administration. The unchanged contezolid recovered from both urine and feces over 168 h accounted for less than 3% of the dose. The DHPO ring opening *via* Baeyer-Villiger oxidation is the main metabolic pathway of contezolid in humans, which generates metabolites M2 and MRX459. The recovery of M2 and MRX459 from urine and feces was approximately 48% and 15% of the dose, respectively (Wu et al., 2021).

Considering the fact that contezolid is mainly metabolized in the liver, it is reasonably expected that hepatic impairment might affect, to some extent, the metabolism and excretion of contezolid. Currently, it is not clear whether it is required to adjust the dose of contezolid in patients with moderate hepatic impairment. For this reason, we designed this study to compare the pharmacokinetics (PK) and safety of contezolid between patients with moderate hepatic impairment and healthy controls with normal liver function after oral administration. Monte Carlo simulation was also performed to assess the necessity for dose adjustment in patients with moderate hepatic impairment based on the PK and pharmacodynamic (PD) data (Chua et al., 2021).

## 2 Methods and materials

### 2.1 Study design and ethics statement

This study was designed as a single-center, open-label, parallel group clinical trial. The study protocol and informed consent form (ICF) were approved by Huashan Hospital Institutional Review Board, Fudan University [No. 2017 (307)]. This clinical trial was conducted in compliance with the International Conference on Harmonization (ICH) Good Clinical Practice guidelines and the principles of the Declaration of Helsinki. All subjects provided their informed consent before participating in the study. This study was registered at chinadrugtrials.org.cn (identifier: CTR20171377).

### 2.2 Study participants

Six patients with moderate hepatic impairment and 6 healthy volunteers (controls) with normal liver function were enrolled to receive a single dose of 800 mg contezolid tablets. All the patients with moderate hepatic impairment satisfied the following criteria: females or males, 18–70 (inclusive) years of age, body mass index (BMI) 17–30 kg/m^2^ (inclusive), diagnosed with moderate hepatic impairment, which was defined as Child-Pugh Category B with a score of 7–9 (significant functional compromise). Liver cirrhosis was identified in all of the patients by ultrasonography, CT scan, magnetic resonance imaging (MRI), or liver biopsy. All patients had an estimated glomerular filtration rate (eGFR) > 50 mL/min/1.73 m^2^ (eGFR was calculated according to CKD-EPI equation which is provided in Supplementary Table S1). Healthy subjects with normal liver function were enrolled as controls in a ratio of 1:1 to match the patients with moderate hepatic impairment in terms of sex, BMI (±15%), and age (±5 years). Healthy status was confirmed in terms of the assessments of medical history, physical examination findings, vital signs, laboratory tests, ultrasonography, and chest radiograph.

The patients or healthy volunteers were excluded if they had any of the following conditions: history of hypersensitivity to contezolid or other oxazolidinone antibiotics; any uncontrolled acute or chronic disease; immunocompromised or under treatment with immunosuppressants; esophageal varices bleeding within 6 months; spontaneous bacterial peritonitis or massive ascites; acute or subacute liver failure; Gilbert syndrome; alanine aminotransferase (ALT) or aspartate aminotransferase (AST) elevation >5 × upper limit of normal (ULN); serum total bilirubin >3 × ULN and alkaline phosphatase (ALP) > 2 × ULN; or international normalized ratio ≥1.5. The patients were also excluded if they had abused alcohol, tabacco, or illegal drugs.

### 2.3 PK sample collection

Blood and urine samples were collected to determine the concentrations of contezolid and its metabolite M2, and compare the PK profiles. The patients and healthy subjects received a single oral dose of 800 mg contezolid within 30 min after a standard meal. Blood samples were collected at predose (within −2 h), and 0.25, 0.5, 1, 1.5, 2, 3, 4, 6, 8, 12, 24, and 48 h postdose. Urine samples were also collected at predose (−12 to 0 h), and 0–4, 4–8, 8–12, 12–24, and 24–48 h intervals postdose, respectively.

### 2.4 PK analysis

Plasma and urine concentrations of contezolid and metabolite M2 were determined by a validated ultra-performance liquid chromatography-tandem mass spectrometry (UPLC-MS/MS) assay (Wang et al., 2022). PK parameters were calculated using non-compartmental analysis (WinNonlin, version 7.0, Certara, Princeton, NJ, United States), including C_max_, the time to C_max_ (T_max_), AUC_0-t_, AUC_0-∞_, elimination half-life (t_1/2_), mean residence time (MRT), clearance (CL/F), apparent volume of distribution (V_z_/F). For each collection time interval, the cumulative amount excreted (Ae) in urine was calculated according to the urine concentration and urine volume. Percent of Ae_0–24h_ in total urinary excretion (Ae_0–24h_%), percent of Ae_0–48h_ in total urinary excretion (Ae_0–48h_%), and renal clearance (CL_R_) were calculated over the time period.

### 2.5 Monte carlo simulation

Non-parametric superposition analysis was conducted to estimate the individual steady-state plasma concentration-time profiles after oral dose of contezolid 800 mg q12h in WinNonlin (version 7.0, Certara, Princeton, NJ, United States). The concentration-time profiles were described with summary statistics. Steady-state PK parameters were calculated. Monte Carlo simulation was performed to determine the probability of target attainment (PTA) and cumulative fraction of response (CFR) against MRSA infections following the recommended dosing regimen of contezolid 800 mg q12h using MATLAB software (version 7.0.1, Mathworks, Inc., United States). Individual PK parameters of 5,000 virtual subjects were generated assuming a log-normal distribution for each PK parameter in both groups. Based on a previous report (Wu et al., 2019), unbound drug AUC_0–24h_/MIC (*f*AUC_0–24h_/MIC) target value of 2.3 was regarded as the PK/PD index best correlating with efficacy in mouse thigh infection models. Clinical isolates of *S. aureus* were collected from 30 hospitals across China during the period from 2015 to 2017 to obtain the distribution of contezolid MIC against MRSA (Wu et al., 2019). The dosing regimen that would achieve a PTA or CFR >90% was considered as optimal for treating infections caused by a specific microorganism.

### 2.6 Safety and tolerability assessments

Safety evaluation was based on treatment emergent adverse events (TEAEs), vital signs, physical examination findings, laboratory tests, and electrocardiograms.

### 2.7 Statistical analysis

Statistical analysis was carried out using SAS software 9.4 (SAS Institute, Cary, NC, United States). Individual subject ratios (moderate hepatic impairment/normal liver function) were obtained from an analysis of variance (ANOVA) with log-transformed AUC or C_max_ as dependent variables using mixed effects model. Moderate hepatic impairment was considered as a fixed effect. Least squares mean difference in log-transformed parameters were back-transformed to calculate the estimated ratio (ER) and the two-sided 90% confidence interval (CI). The AUC_0–t_, AUC_0–∞_, Ae_0–24h_%, and Ae_0–48h_% values of contezolid and M2 was compared between the patients with moderate hepatic impairment and healthy controls using *t*-test at significance level of *p* < 0.05.

## 3 Results

### 3.1 Demographics

Twelve subjects (10 males and 2 females) were enrolled. The mean age (46 vs. 46 ears) and BMI (23.95 ± 4.87 vs. 23.77 ± 3.39 kg/m^2^) were comparable between the patients with moderate hepatic impairment (n = 6) and the controls with normal liver function (n = 6). Other baseline demographic data were generally similar between the two groups. Serum creatinine level did not show significant difference between the two groups (70.5 vs. 68.5 μmol/L, *p* = 0.7806). Hepatic impairment was primarily due to hepatitis B and autoimmune hepatitis. The mean of the Child-Pugh scores in moderate hepatic impaired group is 7 (range: 7–8). All study participants completed the study per protocol (Table 1).

**TABLE 1:** Baseline characteristics compared between the patients with moderate hepatic impairment and healthy controls with normal liver function.

Characteristic	Healthy controls with normal liver function (n = 6)	Patients with moderate hepatic impairment (n = 6)
Sex		
Female	1 (16.7)	1 (16.7)
Male	5 (83.3)	5 (83.3)
Asian race	6 (100)	6 (100)
Age (years)	46 (7.0)	46 (7.3)
Height (cm)	163.55 (7.09)	171.17 (4.36)
Weight (kg)	63.83 (11.75)	70.48 (15.75)
Body mass index (kg/m^2^)	23.77 (3.39)	23.95 (4.87)
Albumin	47.70 (2.50)	32.0 (4.47)
Alanine aminotransferase	17.70 (8.33)	38.80 (13.64)
Aspartate aminotransferase	20.30 (3.20)	45.80 (19.27)
Total bilirubin	11.43 (1.45)	31.42 (20.31)
Serum creatinine	68.50 (12.82)	70.50 (11.34)

Data are presented as number (%) or mean (standard deviation) unless otherwise specified.

### 3.2 PK analysis

**FIGURE 1:**
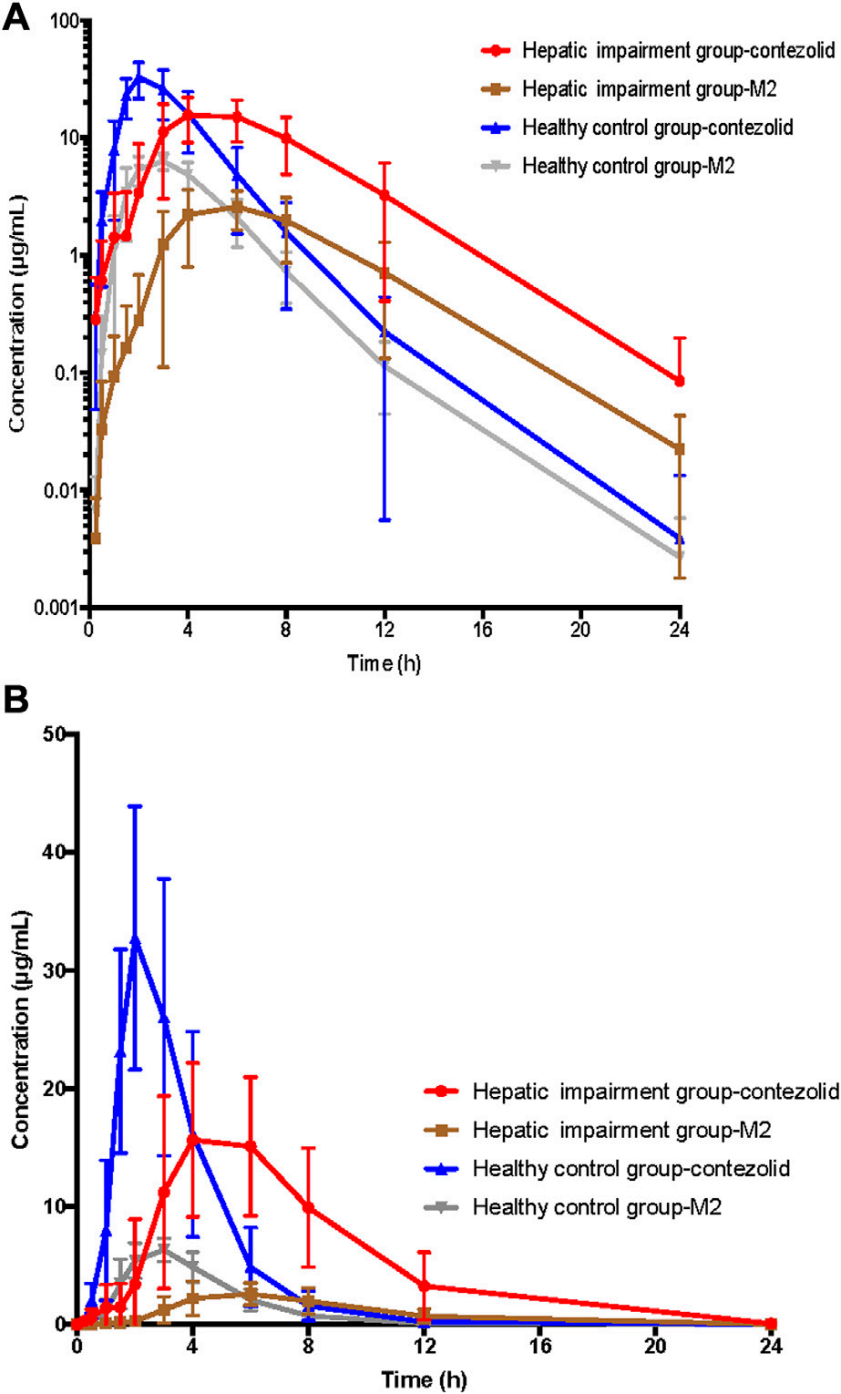
Mean (±SD) contezolid and M2 plasma concentration-time profiles following a single oral dose of contezolid 800 mg in the patients with moderate hepatic impairment and healthy controls with normal liver function. **(A)** The semi-logarithmic scale. **(B)** The linear scale. SD, standard deviation.

The PK parameters of contezolid were provided for the patients with moderate hepatic impairment and the healthy controls (Table 2). The mean (SD) contezolid plasma concentration-time profiles are illustrated in Figure 1. Following a single dose of 800 mg contezolid, the plasma concentration reached peak later in the patients with moderate hepatic impairment than in the healthy controls (median T_max_, 3.98 vs. 2 h). The patients with moderate hepatic impairment had lower C_max_ (geometric mean) than the healthy controls with normal liver function (19.03 vs. 34.49 μg/mL, *p* < 0.05), while the geometric mean value of AUC_0–24h_ was similar between the two groups (106.79 vs. 97.07 h μg/mL). The geometric mean value of terminal elimination half-life (t_1/2_) in the patients with moderate hepatic impairment was longer than that in the healthy controls (1.99 vs. 1.35 h). The oral clearance of contezolid from plasma (CL/F) was similar between the two groups (geometric mean 7.48 vs. 8.24 L/h).

**TABLE 2:** Pharmacokinetic parameters of contezolid in patients with moderate hepatic impairment versus the healthy controls with normal liver function after single oral dose administration of 800 mg contezolid.

PK parameter	Healthy controls with normal liver function (n = 6)	Patients with moderate hepatic impairment (n = 6)	Ratio (90% CI) (%)	*p*-value
C_max_ (µg/mL)	34.49 (25.61)	19.03 (25.78)	55.2 (42.3, 71.9)	0.0022
T_max_ (h)	2.00 (1.50, 3.00)	3.98 (3.00, 6.05)	NA	NA
AUC_0–24h_ (h µg/mL)	97.07 (35.03)	106.79 (34.87)	100.0 (77.1, 156.9)	0.6368
AUC_0–∞_ (h µg/mL)	97.07 (35.05)	107.02 (35.10)	100.2 (77.2, 157.5)	0.6307
λz (/h)	0.51 (26.94)	0.35 (24.48)	NA	NA
t_1/2_ (h)	1.35 (26.94)	1.99 (24.48)	NA	NA
MRT (h)	3.23 (16.45)	6.59 (22.02)	NA	NA
CL/F (L/h)	8.24 (35.05)	7.48 (35.10)	NA	NA
V_z_/F (L)	16.10 (18.23)	21.44 (16.47)	NA	NA

Data are presented as geometric mean (coefficient of variation, %) or median (minimum, maximum) unless otherwise specified. C_max_, maximum concentration; T_max_, time to reach C_max_; NA, not available; AUC_0–24h_, area under the concentration-time curve from time 0–24 h; AUC_0-∞_, area under the concentration-time curve from time 0 to infinity; λz, elimination rate constant; t_1/2_, terminal half-life; MRT, mean residence time; CL/F, oral clearance of drug from plasma; V_z_/F, apparent volume of distribution; CI, confidence interval.

The mean concentration-time profiles of the primary circulating metabolite M2 are also shown in Figure 1. Similarly, the patients with moderate hepatic impairment had lower C_max_ (geometric mean) of M2 than the healthy controls (2.83 vs. 6.63 μg/mL, *p* < 0.05), while the geometric mean AUC_0–24h_ of M2 in the patients with moderate hepatic impairment was slightly lower than that in the healthy controls (18.47 vs. 25.47 h μg/mL, *p* = 0.05) (Supplementary Table S2).

**FIGURE 2:**
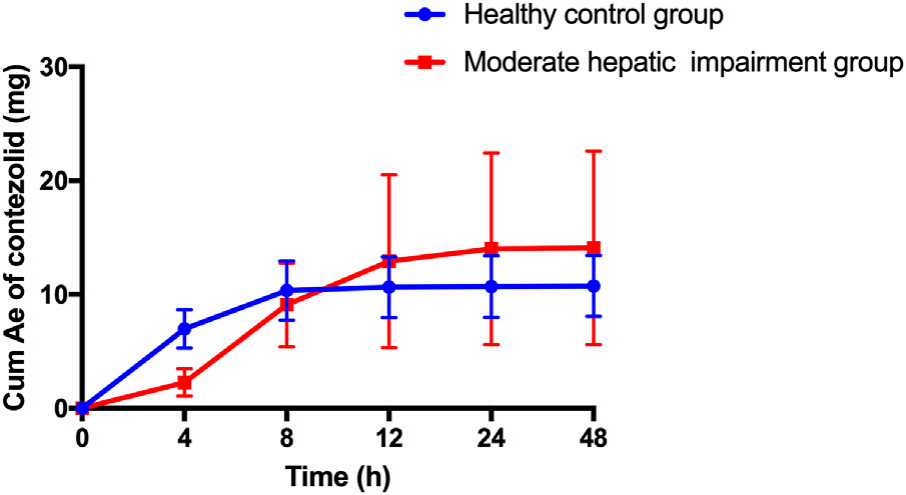
Mean (±SD) cumulative amount of contezolid excreted in urine following a single oral dose of contezolid 800 mg in patients with moderate hepatic impairment and healthy controls with normal liver function. SD, standard deviation; Cum Ae, cumulative amount of contezolid excreted in urine.

The patients with moderate hepatic impairment showed slightly greater mean Ae_0–48h_ and Ae_0–48h%_ of contezolid (Figure 2), but lower mean Ae_0–48h_ and Ae_0–48h%_ of M2 (Supplementary Figure S1) than the healthy controls. The Ae_0–24h_, Ae_0–24h%_, Ae_0–48h_, and Ae_0–48%_ of contezolid and M2 did not show significant difference between the two groups (*t*-test, *p* > 0.05).

The AUC_0–24h_ of contezolid was similar between the patients with moderate hepatic impairment and healthy controls with normal liver function, which was also supported by estimated ratio (ER) of 1.00 (90% CI: 0.77–1.57) (Table 2). Moderate hepatic impairment was associated with lower C_max_ of contezolid compared with the healthy controls with normal liver function (ER 0.55, 90% CI: 0.42–0.72).

### 3.3 PK/PD analysis

**FIGURE 3:**
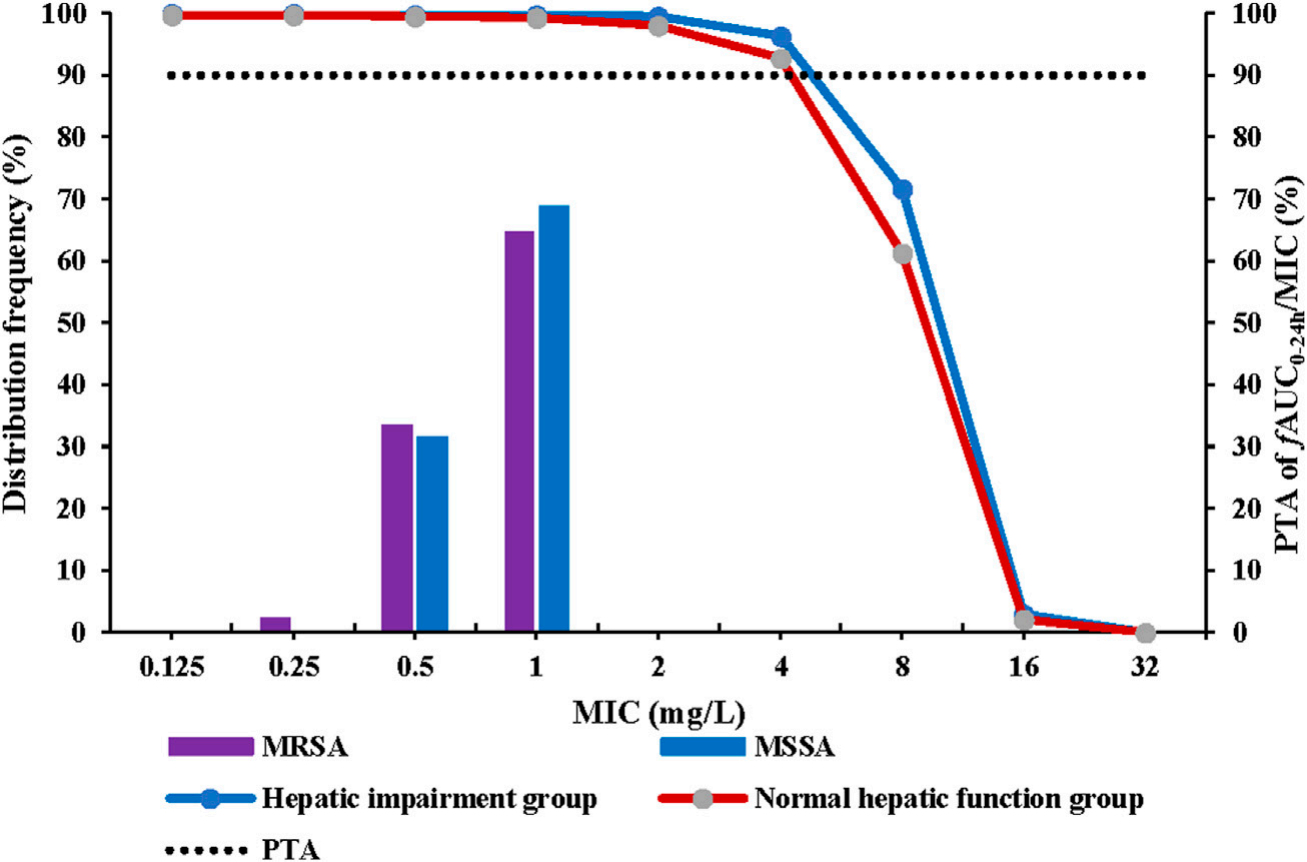
PTA at fAUC_0–24h_/MIC target of 2.3 following oral administration of contezolid 800 mg q12h in the patients with moderate hepatic impairment and healthy controls with normal liver function. MIC, minimum inhibitory concentration; MSSA, methicillin-susceptible *S. aureus*; MRSA, methicillin-resistant *S. aureus*; q12h, every 12 h; PTA, probability of target attainment.

The recommended dosing regimen for contezolid is 800 mg q12h for 7–14 days for treatment of cSSTI, especially the infections caused by MRSA (Hoy, 2021). Non-parametric superposition modeling was conducted to simulate the multiple dose administration (800 mg q12h for 7 days) of contezolid in both groups. Monte Carlo simulation was then performed to evaluate the PTA and CFR for contezolid. According to the simulation results, contezolid 800 mg q12h would be effective for treatment of the infections caused by a MRSA strain when the minimum inhibitory concentration (MIC) is equal to or less than 4 mg/L (PTA >90%) in both groups (Figure 3). According to the previously reported MIC distributions (Wu et al., 2019), the estimated CFR was higher than 99% in both the patients with moderate hepatic impairment (99.8%) and the healthy controls with normal liver function (99.4%) when contezolid was administered at dose of 800 mg q12h against MRSA strains (MIC ≤4 mg/L).

### 3.4 Safety

The overall incidence of TEAEs are summarized in Table 3. Two of the 12 subjects reported at least one TEAE during the study. Five TEAEs (vomiting, nausea, dizziness, proteinuria) were mild in severity. The two TEAEs (direct bilirubin elevation, total bilirubin elevation) were moderate in severity in the patients with moderate hepatic impairment. The direct bilirubin elevation and total bilirubin elevation were reported as serious TEAEs due to a prolonged hospital stay in a subject diagnosed with autoimmune hepatitis. The direct bilirubin and total bilirubin levels were 1.4-fold and 1.6-fold elevation at 48 h post-dose compared to the baseline values, respectively, but recovered to baseline on 11 day. The bilirubin elevation events were due to dose reduction of methylprednisolone and unlikely related to contezolid according to the investigator. Two TEAEs (rash, serum potassium decreased) were mild in severity in subjects with normal liver function. All the TEAEs were self-limiting. Rash occurred in a subject with normal liver function, which was possibly related to contezolid assessed by the investigator. The other TEAEs were assessed by the investigator as not related to contezolid. No AE leading to early discontinuation or death was reported in this study.

**TABLE 3:** Incidence of treatment emergent adverse events after a single oral dose administration of 800 mg contezolid compared between patients with moderate hepatic impairment and healthy controls with normal liver function.

TEAE	Normal liver function (n = 6)	Moderate hepatic impairment (n = 6)	Total (N = 12)
Subjects with at least 1 TEAE	2 (33.3)	2 (33.3)	4 (33.3)
Direct bilirubin elevation	0	1 (16.7)	1 (8.3)
Total bilirubin elevation	0	1 (16.7)	1 (8.3)
Proteinuria	0	1 (16.7)	1 (8.3)
Serum potassium decreased	1 (16.7)	0	1 (8.3)
Dizziness	0	1 (16.7)	1 (8.3)
Rash	1 (16.7)	0	1 (8.3)
Nausea	0	1 (16.7)	1 (8.3)
Vomiting	0	1 (16.7)	1 (8.3)

Data are expressed as number (%) unless otherwise specified. TEAE, treatment emergent adverse event.

## 4 Discussion

The liver plays a central role in the metabolism and elimination kinetics of most drugs and their active or non-active metabolites (Flanagan et al., 2014). The United States Food and Drug Administration guidance recommends PK studies in patients with impaired hepatic function if >20% of the absorbed dose of a drug is eliminated by the liver. Majority of the administered contezolid dose (>97%) is metabolized in the liver (Wu et al., 2018). Therefore, it is required to clarify whether the dose of contezolid should be adjusted in patients with moderate hepatic impairment because contezolid may be prescribed as an alternative antimicrobial therapy in such patients.

Our preliminary data indicated that moderate hepatic impairment did not affect the drug exposure (AUC) after a single oral administration of 800 mg contezolid under fed condition compared with normal liver function volunteers even though lower C_max_ in the patients with moderate hepatic impairment. The unbound drug AUC to MIC (*f*AUC/MIC) ratio was recognized as the best PK/PD index for predicting the clinical efficacy of contezolid. According to Monte Carlo simulation results, at the proposed AUC/MIC target value of 2.3, contezolid 800 mg q12h could achieve satisfactory PTA and CFR (both >90%) in subjects with moderate hepatic impairment. Meanwhile, the results of PK/PD analysis for the subjects with normal liver function were consistent with the results in a previous study (Wu et al., 2019), which further supports the reliability of our simulation results for the patients with moderate hepatic impairment. Therefore, it is not required to adjust the dose of contezolid in patients with moderate hepatic impairment.

In a previous Phase I, single-center, three-part, randomized study evaluating the PK and safety of single and multiple oral doses of contezolid in healthy Chinese adults, contezolid was rapidly absorbed after oral administration. The t_1/2_ was approximately 2.8–4.8 h when the drug was taken with food. About 2% of the administered dose of contezolid was excreted *via* kidneys in unchanged form (Wu et al., 2018). A previous clinical study in healthy Chinese subjects showed that the mean C_max_ and AUC_0–23.5h_ of contezolid was 26.5 μg/mL and 96.8 h μg/mL after a single oral dose of 800 mg contezolid under fed condition. The results of the healthy subjects with normal liver function in this study are consistent with the results of previous PK study, which reinforces the validity of this study (Wu et al., 2019).

**FIGURE 4:**
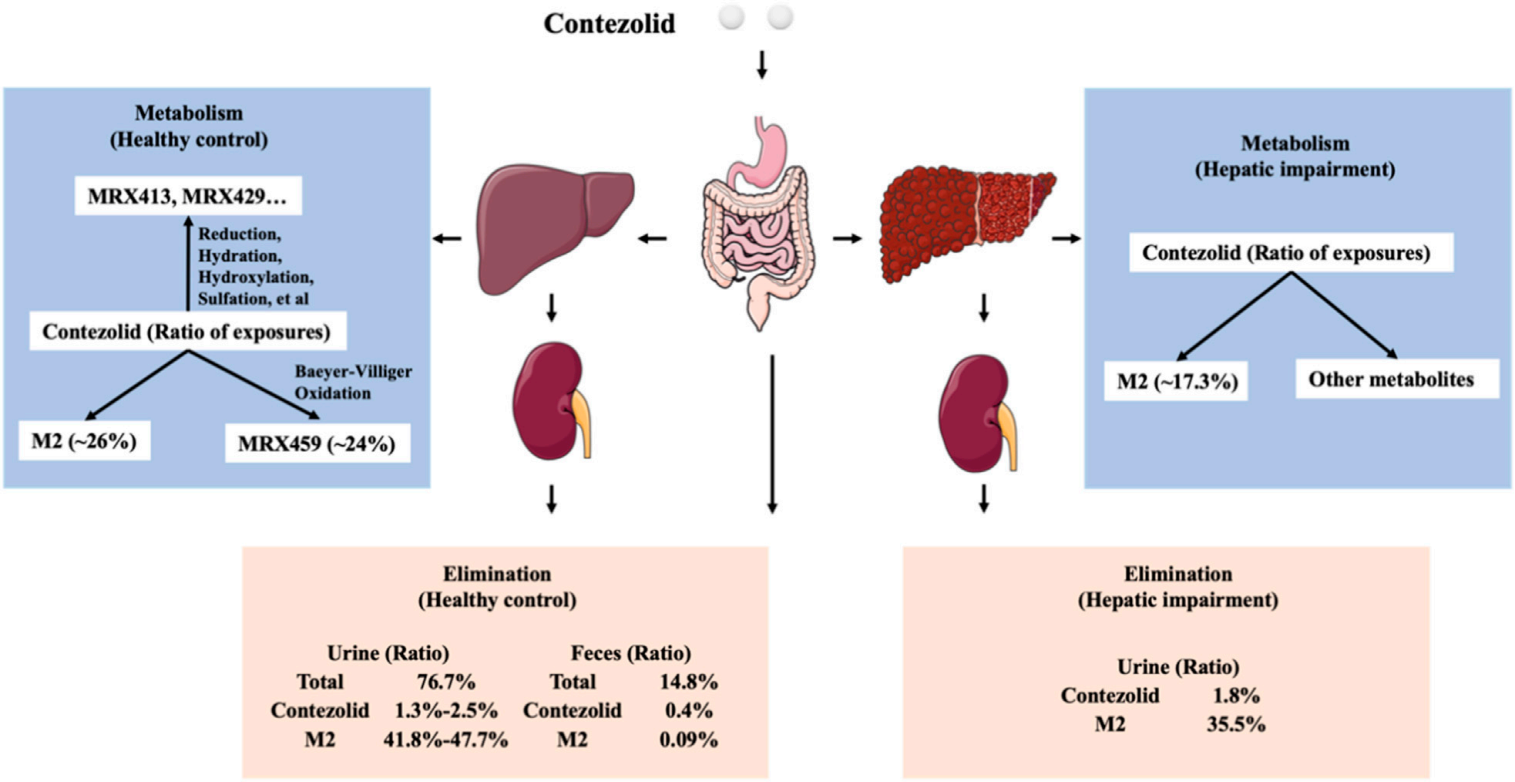
Comparison of metabolism and elimination of contezolid in terms of PK parameter ratios between the patients with moderate hepatic impairment and healthy controls with normal liver function.

As demonstrated by the concentration-time curves, the patients with moderate hepatic impairment showed lower C_max_ associated with a significantly longer T_max_ (3.98 vs. 2 h) compared to the healthy controls. However, the AUC_0–∞_ of contezolid did not show significant difference between the two groups (107.02 vs. 97.07 h μg/mL, *p* = 0.63). Other PK parameters were also comparable between the two groups. Most of the PK parameters of M2 were also similar between the patients with moderate hepatic impairment and healthy controls, except AUC_0–24h_ of M2, which was significantly lower in the patients with moderate hepatic impairment (18.47 vs. 25.47 h μg/mL, *p* = 0.05). The metabolism and elimination of contezolid were compared in terms of the PK parameter ratios between the patients with moderate hepatic impairment and healthy controls based on several previous studies (Figure 4 **)** (Meng et al., 2015; Wu et al., 2018; Wu et al., 2019; Wu et al., 2021), which highlights the main metabolism and elimination pathways of contezolid. In our study, the mean plasma exposure of M2 following a single oral dose of 800 mg contezolid was 17.3% in the patients with moderate hepatic impairment and 26.2% in healthy controls. The ratio of total cumulative urinary excretion (contezolid and M2 which was converted to contezolid) between the two groups was 37.3% and 43.2%, respectively, which suggests that moderate hepatic impairment does not have significant effect on the metabolism and elimination of contezolid. The change of cumulative amount excreted in urine was consistent with the differential exposure of contezolid between the two groups. Moderate hepatic impairment was associated with slightly higher AUC of contezolid, but lower exposure of M2 compared to the healthy controls, which indicates lower metabolic change of contezolid in patients with moderate hepatic dysfunction. Another oxazolidinone, tedizolid, is eliminated mainly by hepatic excretion *via* bile. About 80% of the administered dose is eliminated in feces. Both contezolid and tedzolid exhibit high protein binding (90% vs. 80%). Patients with moderate or severe hepatic impairment presented approximately 22% and 34% higher AUC_0–∞_, respectively compared with those in the control group after a single oral dose of 200 mg tedizolid phosphate. Therefore, no dose adjustment is required for tedizolid phosphate in patients with any degree of hepatic impairment (Flanagan et al., 2014). Similarly, a lower exposure change of the contezolid was observed in patients with moderate hepatic impairment compared to healthy controls, suggesting that contezolid AUC might not increase significantly in patients with severe hepatic impairment.

Gastrointestinal dysfunction has been described in patients with liver disease, which may affect the absorption of orally administered drugs (Verbeeck, 2008). In the population PK studies conducted by Li et al. (2020) and Yuan et al. (2022) in healthy volunteers and patients, PK variability of contezolid was heavily affected by body weight, food effect, and disease status rather than liver or renal function. Disease status could significantly affect the absorption rate and peripheral volume of distribution. The lower C_max_ and prolonged T_max_ in the patients with moderate hepatic impairment may be explained by the slower absorption rate of contezolid in cirrhotic patients compared with the healthy controls with normal liver function. Yuan et al. (2022) reported that food, subject type, and body weight were significant covariates for the PK parameters of contezolid. In these two studies, body weight was a significant covariate for V2 and CL, and the effect on the apparent volume of distribution is more notable. In the present study, body weight did not show significant difference between the two groups (*p* > 0.05). Therefore, the change of contezolid exposure was not due to body weight.

Liver disease is usually associated with extensive genetic regulation of cytokines and drug-metabolizing enzymes (Dietrich et al., 2016; El-Khateeb et al., 2021). However, non-CYP and non-UGT enzymes did not show significant difference in their relative distribution between the healthy controls and patients with liver disease (El-Khateeb et al., 2021). Through incubation of human liver microsomes, cytosol, and S9 fractions, liver cytosol-fortified recombinant flavin-containing monooxygenases (FMOs), and human hepatocytes in the presence of contezolid, the enzymes involved in the metabolism of contezolid were identified as FMOs, aldo-keto reductase (AKR), short-chain dehydrogenase/reductase (SDR), aldehyde dehydrogenase (ALDH), and aldehyde oxidase (AO), but no CYP enzymes were involved (Meng et al., 2015). The reduction in the expression of metabolizing enzymes is typically associated with reduced drug clearance and increased AUC. The mean level of albumin was 32.0 g/L (95% CI: 27.31, 36.69) in the patients with moderate hepatic impairment and 47.7 g/L (95% CI: 45.04, 50.29) in healthy controls in the present study. Serum creatinine level did not show significant difference between the two groups. The decreased AUC and cumulative urinary excretion of M2 in patients with moderate hepatic impairment may support the hypothesis that moderate hepatic impairment has minimal effect on the metabolism of contezolid in the liver.

The single-dose study design is a limitation of this study. This was based on the following considerations. Firstly, contezolid demonstrated linear PK when the dosage was not higher than 800 mg, and no accumulation was found after multiple doses of contezolid (Wu et al., 2018; Wu et al., 2019). Secondly, the single-dose PK study for contezolid in patients with moderate hepatic impairment may be satisfactory according to the guidance for industry on the evaluation of the PK of medicinal products in the patients with impaired hepatic function issued by FDA (Guidance, 2003). The unbound concentration of contezolid was not determined. This is also a limitation of this study. However, albumin concentration was lower in moderate hepatic impairment group, which might be associated with higher unbound contezolid concentration because of high plasma protein binding of contezolid, which may represent higher *f*AUC_0–24h_/MIC to treat the target pathogen.

In the population PK studies mentioned previously, liver function, and renal clearance did not exert significant impact on the PK behavior of contezolid, which may also support the result that moderate hepatic function impairment has minimal effect on the PK of contezolid. Viral infections may have some effect on drug metabolism. Some of the patients included in this study had viral hepatitis, but they were receiving stable treatment. Those with acute or subacute hepatic failure, or discontinuing antiviral drug within 1 year were excluded. The patients included in this study had a stable and moderate hepatic impaired liver disease. Viral infection was not considered a significant confounding factor in this study. Our study population was also consistent with the study population recommended in the Guideline on the Evaluation of the Pharmacokinetics of Medicinal Products in Patients with Impaired Hepatic Function” from European Medicines Agency (Guideline, 2023). It is important to note that only moderate hepatic impaired patients were included in this study, caution should be exercised when prescribing contezolid for the patients with severe hepatic impairment. In addition, the small sample size in this study may weaken the conclusion. The data should be interpreted cautiously and the safety of contezolid will be further evaluated in more hepatic impaired patients after treatment with the recommended dosing regimen in post-marketing safety surveillance.

## 5 Conclusion

Our study demonstrated that the AUC of contezolid was similar between the patients with moderate hepatic impairment and the healthy controls with normal liver function, even though significantly lower C_max_ in the patients with moderate hepatic impairment. Ae_0–48h_ and CL_R_ of contezolid did not show significant difference between the two groups. Monte Carlo simulation results support that at the proposed AUC/MIC target value of 2.3, the dosing regimen of contezolid 800 mg q12h could achieve satisfactory PTA and CFR (both >90%) in patients with moderate hepatic impairment. Therefore, dose adjustment is not required for contezolid in case of moderate hepatic impairment.

## Data Availability

The raw data supporting the conclusion of this article will be made available by the authors, without undue reservation.
