# Supplementary material for: Dose adjustment not required for contezolid in patients with moderate hepatic impairment based on pharmacokinetic/pharmacodynamic analysis
Source: Front Pharmacol. 2023 Mar 13;14:1135007. doi: 10.3389/fphar.2023.1135007 (PMC10040594; doi:10.3389/fphar.2023.1135007)
Supplement: Supplementary file 1 [file DataSheet1.docx]

**Supplementary Material**

**Title:**

**Dose Adjustment Not Required for Contezolid in Patients with Moderate Hepatic Impairment Based on Pharmacokinetic/Pharmacodynamic Analysis**

**Supplementary Table 1.** The CKD-EPI equation for estimating GFR.

| **Sex** | **Serum Creatinine**  **μmol/L (mg/dL)** | **Equation** |
| --- | --- | --- |
| Female | ≤62 (≤0.7) | eGFR = 144 × (Scr/62(0.7))^-0.329^ × (0.993)^Age^ |
|  | >62 (>0.7) | eGFR = 144 × (Scr/62(0.7))^-1.209^ × (0.993)^Age^ |
| Male | ≤80 (≤0.9) | eGFR = 144 × (Scr/80(0.9))^-0.411^ × (0.993)^Age^ |
|  | >80 (>0.9) | eGFR = 144 × (Scr/80(0.9))^-1.209^ × (0.993)^Age^ |
|  | |  |

**Supplementary Table 2.** Pharmacokinetic parameters of metabolite M2 in patients with moderate hepatic impairment and the healthy controls with normal liver function after single oral dose administration of 800 mg contezolid.

| **PK parameter** | **Healthy controls with normal liver function**  **(n=6)** | **Patients with moderate hepatic impairment**  **(n=6)** |
| --- | --- | --- |
| C_max_ (µg/mL) | 6.63 (14.03) | 2.83 (44.87) |
| T_max_ (h) | 3.00 (2.00, 3.00) | 5.98 (4.00, 8.00) |
| AUC_0-24h_ (h∙µg/mL ) | 25.47 (20.12) | 18.47 (30.15) |
| AUC_0-∞_ (h∙µg/mL) | 25.48 (20.15) | 18.56 (29.94) |
| λz (1/h) | 0.41 (22.19) | 0.30 (20.34) |
| t_1/2_ (h) | 1.70 (22.19) | 2.34 (20.34) |
| MRT (h) | 4.05 (14.73) | 7.49 (21.00) |

Data are presented as geometric mean (coefficient of variation, %) or median (minimum, maximum) unless otherwise specified.

*C_max_, maximum concentration; T_max_, time to reach C_max_; AUC_0-24h_, area under the concentration-time curve from time 0 to 24 h; AUC_0-∞_, area under the concentration-time curve from time 0 to infinity; λz, elimination rate constant; t_1/2_, terminal half-life; MRT, mean residence time.*


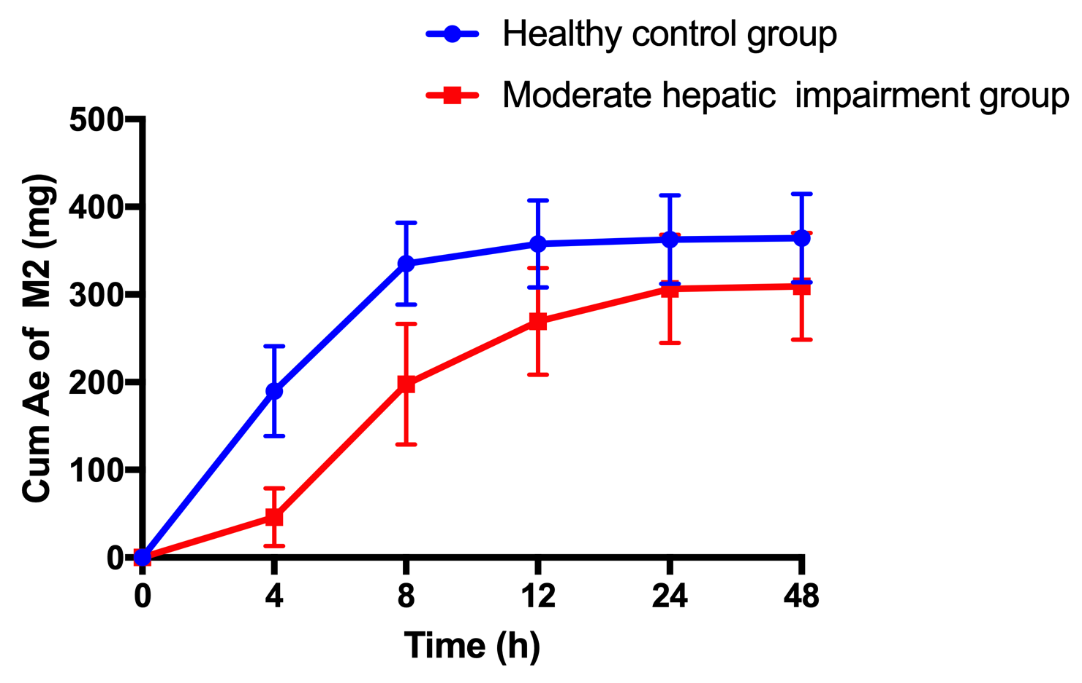


**Supplementary Figure 1.** Mean (± SD) cumulative amount of M2 excreted in urine following a single dose oral administration of contezolid 800 mg in patients with moderate hepatic impairment and the healthy controls with normal liver function. SD, standard deviation; Cum Ae, cumulative amount of M2 excreted in urine.
